# Latent profile analysis of depression in older adults spouse caregivers

**DOI:** 10.3389/fpubh.2025.1450720

**Published:** 2025-03-11

**Authors:** Fuzhe Feng, Yalong Huang

**Affiliations:** ^1^School of Medicine, Sias University, Henan, China; ^2^The Third People’s Hospital of Henan Province, Zhengzhou, China

**Keywords:** depression, older spouse caregivers, latent profile analysis, related factors, older

## Abstract

**Objective:**

With advancing age, older adults are more likely to experience health problems and a decline in functioning, necessitating long-term care. Spouses play a crucial role in providing care for the older adults. Depression is a significant mental health issue faced by older adult’s spouses. Categorizing depression into homogeneous subgroups can unveil hidden insights.

**Methods:**

This study utilized the Harmonized CHARLS dataset to investigate. Latent Profile Analysis (LPA) was employed to identify subgroups of older adult’s spouses who experience depression, and chi-square tests were conducted for univariate analysis. Furthermore, multiple logistic regression was utilized to analyze the associated factors.

**Results:**

Spouse caregivers were identified and classified as Low Level Depression (50.6%), High Level Depression (20.0%), and Unstable Affective Depression (29.4%). Gender, education level, self-assessment of health, communication with children, social participation, life satisfaction, and place of residence were found to be influential factors for depression among older adults spouse caregivers. Multiple logistic regression analysis indicated that, compared to individuals with low levels of depression, those with high levels were significantly associated with gender, education level, self-assessed health status, social engagement, life satisfaction, and place of residence. Similarly, compared to individuals with low levels of depression, those classified as having an unstable affective type were significantly associated with gender, education level, self-assessed health status, and life satisfaction. Furthermore, compared to individuals with high levels of depression, those with unstable affective depression were significantly associated with gender, self-assessed health status, weekly interactions with children, and participation in social activities.

**Conclusion:**

This study revealed distinct subtypes of depression among older adults spousal caregivers, emphasizing the importance of targeted interventions in primary care. Tailored intervention strategies addressing the specific characteristics of each subtype may improve caregivers’ mental health and enhance their quality of life.

## 1 Introduction

The global older adult’s population is experiencing a significant growth both in terms of numbers and proportions. From 2020 to 2030, the global population of individuals aged 60 and above is projected to rise from 1 billion to 1.4 billion, indicating a substantial increase of 34%. This aging trend is occurring at a considerably faster pace compared to previous periods. By 2050, two-thirds of the world’s population aged 60 and older will live in low- and middle-income countries (1). In China alone, projections indicate that by 2050, approximately 400 million individuals will be aged 65 or older, with an additional 150 million over the age of 80 (2). As global aging continues, the associated challenges in older adults care are intensifying, particularly in low- and middle-income countries where medical resources and infrastructure are limited. These constraints further increase the demand and burden of older adults care in these regions.

Alongside the intensifying global aging trend, rapid economic development and lifestyle changes have led to a significant shift in the global disease profile, with chronic non-communicable diseases (NCDs) now surpassing infectious diseases as the primary threat to human health. In developing countries, the combined pressures of an aging population and the burden of disease are further intensified by limited medical resources and infrastructure, heightening the demand for older adults care (3). In China, non-communicable diseases (NCDs) now account for the largest proportion of the disease burden, responsible for approximately 90% of total deaths in 2019 (4). Due to the complex causes and long progression of chronic diseases, individuals may suffer from multiple chronic conditions simultaneously, resulting in more severe health crises (4) and more complex long-term care needs.

As people age, they are more likely to encounter health challenges and experience functional decline, necessitating long-term care support (5). The concept of “aging in place” has recently garnered increasing attention. In simple terms, it refers to individuals growing older within their own homes. The WHO Center for Health Development defines this concept as “supporting the desire and ability of individuals to live relatively independently within their current home or in appropriately supported housing within the community, through the provision of adequate services and assistance (6)”. Many older adults individuals prefer to spend their later years in familiar surroundings rather than transitioning into institutional care (7). Additionally, the rising costs associated with long-term care (8) and the growing preference for aging within one’s own home (9) have led to a greater emphasis on family caregiving across various countries, with particular attention to spousal caregiving. Research indicates that when older adults face functional impairments, their spouses frequently assume the primary caregiver role (10). Additionally, declining birth rates and increased migration have resulted in smaller family sizes, causing a greater dependency of older adults on their spouses for caregiving (11). This phenomenon is even more pronounced in China, where limited formal caregiving resources and cultural traditions contribute to the prevalent practice of aging in place. Among older adults requiring caregiving assistance, the majority rely on family members, primarily spouses, followed by children and other relatives. In essence, spouses play a crucial role in the provision of elder care (12).

Extensive research has indicated that spousal caregivers, compared to non-spousal caregivers, tend to receive less support from other family members (13–16). They also experience elevated levels of stress and have poorer physical and mental health (17–19). Unlike other older adults, spousal caregivers have a distinctive role in caring not only for their own well-being but also for their spouses’ needs. This caregiving lifestyle appears to render them more susceptible to depression. Studies have shown that providing long-term care directly influences the prevalence of depression among family caregivers (20). Spousal caregivers exhibit higher levels of depressive symptoms compared to non-spousal caregivers (21). Depression, a common psychiatric disorder characterized by cognitive, emotional, and physical symptoms, has become the second leading cause of disability worldwide as estimated by the World Health Organization in 2020. In 2015, over 300 million people worldwide were diagnosed with depression, representing 4.4% of the global population and establishing it as the primary contributor to the global disease burden (22). Among those affected, 5.7% were aged 60 years or older (23). Additionally, the prevalence of depression among the older adults has been increasing; in 2018, the incidence of depression among older adults in China reached a significant 44.5% (24). Depression carries significant economic implications and represents a critical global public health concern. It not only affects the quality of life of older adults’ individuals (25) but also contributes to adverse outcomes such as falls (26), frailty (27), dementia (28), and suicide (29). Older adults already face a high risk of depression, and spousal caregivers constitute a particularly vulnerable subgroup. Therefore, an in-depth analysis of depression among spousal caregivers is essential.

Previous research on spousal depression has focused on longitudinal predictors of depression in spousal caregivers with dementia (30), and the relationship between depression in men as spousal caregivers and other factors (21). However, these studies often diagnose depression in older adults based on total scores or critical values, overlooking the potential qualitative differences in response patterns among individuals who share similar scores. This can lead to significant heterogeneity within defined groups. However, this issue can be addressed through the application of LPA/LCA. LPA/LCA utilizes probabilistic models for classification while simultaneously examining subjects on an individual basis, thereby intuitively revealing group heterogeneity.

In recent years, several studies have utilized the LPA/LCA approach to explore the characteristics of depression in older adults. For example, In 2023, Hou (31) conducted a latent profile analysis (LPA) on older adults living alone, categorizing them into three subgroups based on their depression levels: low (30.4%), moderate (55.3%), and high (14.4%).The study suggested that targeted interventions should be implemented based on the specific depressive conditions of older adults living alone. In 2017, Ni (32) classified depression in older adults into three subgroups: “mild depression,” “severe depression,” and “lack of positive affect.” Their findings revealed varying treatment effects among baseline depression categories, suggesting that tailored intervention plans could be beneficial in improving depression outcomes in older adults. These studies found that the LPA/LCA method can clearly identify homogeneous subgroups of depression, thus providing a better understanding of the differences between different subgroups of depression in older adults.

However, none of these studies specifically analyzed spousal caregiver depression, making it difficult to understand its unique characteristics. While it can be hypothesized that different depressive symptom categories also exist within the spousal caregiver population, their distinct lifestyle characteristics may result in a classification that differs from existing depression studies. The dual role of spousal caregivers—as both caregivers and older adults—presents unique challenges that necessitate different treatment approaches. While the general older adult’s population may benefit from standard mental health interventions, spousal caregivers require more targeted and comprehensive treatment plans to address both their caregiving responsibilities and the personal stress associated with aging. Therefore, previous findings cannot serve as the foundation for interventions aimed at spousal caregivers who are at high risk for depression. Therefore, this study aimed to reveal the categories and influencing factors of depressive symptoms among older spousal caregivers in a Chinese cultural context using a latent profile analysis.

## 2 Methods

### 2.1 Sample

The data used in this study were sourced from the China Health and Retirement Longitudinal Study (CHARLS), a comprehensive national survey designed to gather detailed information on health and aging among individuals aged 45 and older in China. The data encompassed 28 provinces, cities, and autonomous regions across China, ensuring a highly representative sample. To ensure the relevance of the data, certain criteria were applied for screening purposes. The data were obtained from Harmonized CHARLS, a streamlined version of the CHARLS survey providing convenient access to a subset of interview data (33). Specifically, the study focused on older adults aged 60 years and above who reported receiving assistance from their spouses for daily living. Sample losses and missing values were carefully excluded to ensure the integrity of the main outcome variable. After these exclusions, a final sample of 2,224 participants was obtained. The study adhered to the ethical guidelines set forth by the World Medical Association’s code of ethics (Declaration of Helsinki) regarding experiments involving human subjects. It underwent ethical review and approval by the Biomedical Ethics Review Committee of the Local University. Prior to their participation, all participants were provided with comprehensive information and gave their informed consent to be part of the study. Access to the CHARLS data was obtained following the designated regulations, and data were acquired and utilized with approval from the database administrator.

### 2.2 Measurements

#### 2.2.1 Depression

In this study, the CESD-10 was used to score the depression level of older adults spouse caregivers. The Chinese version of this scale has been widely used in numerous studies, demonstrating high reliability and validity, with a Cronbach’s alpha coefficient of 0.802 (34). It includes three factors: depressive mood, somatic retardation, and positive affect. The scale is scored on a 4-point Likert scale, with a score of 1 for “not or rarely,” 2 for “sometimes or a little of the time,” 3 for “often or half of the time,” and 4 for “most or all of the time.” The higher the score, the more severe the depression. In this study, the reverse scoring of the items, including the item “I am hopeful about the future” and the item “I am happy,” was reversed in the LPA.

#### 2.2.2 Identification of spouse caregivers

Within the comprehensive CHARLS dataset, a specific question was included to determine whether the participant’s spouse provided assistance with various activities of daily living. These activities encompassed tasks such as: dressing, bathing, eating, getting in and out of bed, using the toilet, and performing household chores. If a participant indicated that their spouse served as the primary caregiver, their spouse was identified as the spousal caregiver for the purposes of this study.

#### 2.2.3 Covariates

This study also explored the effects of gender, family location, frequency of communication with children, financial support from children, social activities, chronic diseases, smoking, alcohol consumption, life satisfaction, and self-rated health status on older spouse caregivers. Covariate data included (1) family location (urban/rural); (2) financial support from children (yes/no); (3) social activities (yes/no). (4) chronic diseases, including 12 chronic diseases such as hypertension, cataracts, bronchitis/emphysema/asthma or pneumonia (with any of them/without); (5) smoking (yes/no); (6) alcohol (yes/no); (7) whether they are still working now (Yes/No); (8) whether they are retired (Yes/No); (9) self-assessed health status (Very good/Good/Fair/Poor/Very poor); (10) life satisfaction (Not at all satisfied/Not very satisfied/Somewhat satisfied/Very satisfied/Completely satisfied); (11) Gender (Male/Female); (11) Educational attainment (defined using ISCED-97 1. Less than lower secondary education, 2. Upper secondary & vocational training, 3. Tertiary education.).

### 2.3 Statistical analysis

This study mainly used latent profile analysis for data analysis. Unlike variable-centered approaches, LPA aims to identify distinct patterns of multiple variables that occur consistently across individuals rather than focusing solely on individual variables or their interactions (35). By doing so, LPA classifies individuals within heterogeneous populations into smaller, more homogeneous subgroups (36), revealing hidden information that these subgroups bring to light (37). First, a well-fitting latent profile model was explored using the 10-item self-assessment results of the CES-D as an index of exogenous response; again, multinomial logistic regression was used to analyze the factors associated with depression. The above analyses were completed by Mplus 8.2with SPSS 25.0 software. The main evaluation indexes of the latent variable model were AIC, BIC, aBIC, Entropy, LMR, and BLRT. Among them, the smaller the values of AIC, BIC, and aBIC, the better the model fit, Entropy is an index to evaluate the accuracy of category classification, which takes the value of 0 ~ 1. Entropy≥0.8 indicates that the classification accuracy exceeds 90%. lMR and BLRT are used to compare the fit difference between k-1 and k-category models, and the *p*-value of both reaches a significant level indicating that the k-category model is better than the k-1 category model (38–40).

## 3 Results

### 3.1 Sample characteristics

In the present study, we included 2,224 participants for the survey. Of these, 52.5% were female and their mean age was 68.53 years (SD = 5.928). 74.6% of the survey participants lived in rural areas, 93.9% had primary education, 5.5% had secondary education, while only 0.6% had higher education. It is noteworthy that the majority of older people (63.8%) do not participate in exercise and only 3.6% consider themselves in very good health. However, it is encouraging that 30.3% of individuals exhibit a high level of life satisfaction. In addition, our survey revealed other interesting findings. Of these, 1958 participants (88.0%) reported having received financial support from their children, and 806 (36.2%) participated in social activities with others. Also, 2081 (93.6%) had been diagnosed with a chronic disease, 552 (24.8%) had smoked, and 1,018 (45.8%) had consumed alcohol. Detailed data are presented in Table 1.

**Table 1:** General characteristics of older adults spouse caregivers.

Variables	Total [*n* = 2,224; n (%)]	Variables	Total [*n* = 2,224; n (%)]
Age, mean ± SD	68.53(5.928)	Economic supports from children	
Gender		No	266 (12.0)
Male	1,056(47.5)	Yes	1958 (88.0)
Female	1,168(52.5)	Social activities	
Education		No	1,418 (63.8)
1	2088(93.9)	Yes	806 (36.2)
2	122(5.5)	currently working	
3	14(0.6)	No	1,210 (54.4)
Self-report of health		Yes	1,014 (45.6)
Very good	80(3.6)	whether retired	
Good	93(4.2)	No	1,057 (47.5)
Fair	765(34.4)	Yes	1,167 (52.5)
Poor	906(40.7)	Life satisfaction	
Very poor	380(17.1)	Not at all satisfied	104 (4.7)
Drink		Not very satisfied	236 (10.6)
No	1,206(54.2)	Somewhat satisfied	1,119 (50.3)
Yes	1,018(45.8)	Very satisfied	674 (30.3)
Smoke		Completely satisfied	91 (4.1)
No	1,672(75.2)	Chronic diseases	
Yes	552(24.8)	No	143 (6.4)
Contact with children in person/phone/email		Yes	2081 (93.6)
No	272(12.2)	Place of residence	
Yes	1952(87.8)	Urban	566 (25.4)
		Rural	1,658 (74.6)

### 3.2 Identification of older adults spouse caregivers subgroups

Table 2 presents the data for the six fitted models. As the number of categories increases, the AIC, BIC, and aBIC values gradually decrease, while the entropy values all exceed 0.8. Among these models, the four-category model presents the highest entropy value. This indicates that the four-category model may be the most appropriate choice. However, it should be noted that there are certain profiles with a low percentage of less than 0.1 in the four-category and five-category models. In addition, depending on the actual situation, increasing the number of categories may lead to the dispersion of valid information, which may affect the accuracy of the results. As a result, the three-category model was selected as the ultimate fitted model. Table 3 displays the average probability of each subgroup belonging to their respective profiles, ranging from 93.7 to 99.6%. These values indicate the reasonableness of the three potential profile models.

**Table 2:** Indicators for each latent profile of depression in older adults spouse caregivers.

Profile	AIC	BIC	aBIC	Entroy	LMR (P)	BLRT (P)	Proportion
1	70850.84	70964.98	70901.43				
2	66485.78	66662.7	66564.2	0.857	0.000	0.000	0.56835/ 0.43165
3	64608.63	64848.32	64714.88	0.915	0.000	0.000	0.50629/ 0.19964/0.29406
4	64191.9	64494.37	64325.98	0.939	0.000	0.000	0.48831/0.08678/0.25090/0.17401
5	62902.89	63268.14	63064.81	0.938	0.000	0.000	0.09263/ 0.43121/0.21628/0.15108/0.10881
6	62976.86	63404.89	63166.61	0.937	0.937	1	0.18705/0.11691/0.05531/0.32509/0.17055/ 0.14369

AIC, Akaike information criterion; BIC, Bayesian information criterion; aBIC, sample size adjusted BIC; LMR, Lo–Mendell–Rubin likelihood ratio test; BLRT, Bootstrapped likelihood ratio test.

**Table 3:** Attribution probabilities for each latent profile of subjects.

Class	Profile 1	Profile 2	Profile 3
Profile1	0.961	0.001	0.038
Profile2	0.004	0.996	0.000
Profile3	0.063	0.000	0.937

### 3.3 Naming of latent profile

**Figure 1:**
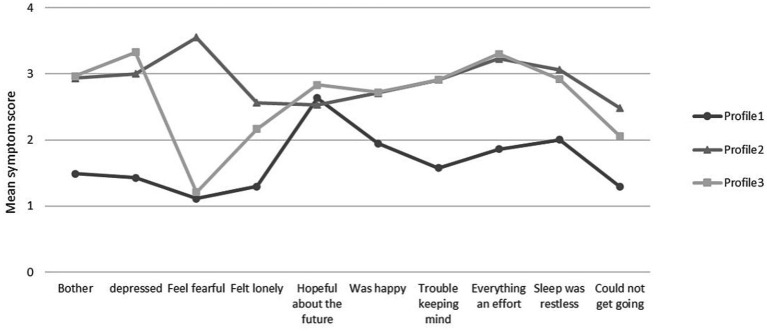
Latent profile model of depression.

Based on the results of the latent profile analysis, we show in Figure 1, the scores of the 3 categories on the 10 entries of the CESD. To enhance clarity, we have arranged the items based on their respective dimensions. The first four entries represent Depressed Affect, the fifth and sixth entries represent Positive Affect, and the seventh to tenth entries represent Somatic Retardation. By looking at the chart, it is clear that the first category scores significantly lower than the second and third categories on each item and that the scores fluctuate less. Based on the characteristics of the scores, we named this profile as “Low Level Depression.” In contrast, the second category scores were significantly higher than the first and third categories, and the scores fluctuated less. Based on its score characteristics, we rated this profile as “high level depression.” As for the third category, its score was not significantly different from the second category, but fluctuated more in the Depressed Affect dimension. Based on the characteristics of its score, we named this profile as “Unstable Affective Depression.”

### 3.4 Inter-profile characteristic differences

Table 4 compares the differences in demographic characteristics across the categories. When comparing all variables in the three categories, *p*-values were less than 0.05 for all variables except for the variables of financial support from children, currently working or not, retired or not, and chronic illness. In all three categories, those who were mostly: female, lived in rural areas, had primary education, did not smoke, did not drink alcohol, and did not participate in social activities were classified as high level depressed, accounting for a total of 20.0%. Notably, those older adults with good health and high life satisfaction were more likely to be in the low level depression type, while those in the high level depression type not only had poorer health and life satisfaction, but also had the lowest frequency of socially engaged activities and interactions with their children.

**Table 4:** Inter-profile characteristic differences.

Variables	Low-level depression *n* = 1,126 [50.6%; n (%)]	High-level depression *n* = 444 [20.0%; n (%)]	Unstable-affective depression *n* = 654 [29.4%; n (%)]	X^2^/F	*p*
Age, mean ± SD	68.94(6.126)	68.33(5.570)	67.95(5.769)	6.152	0.002
Gender				19.053	0.000
Male	593(52.7)	158(35.6)	305(46.6)		
Female	533(47.3)	286(64.4)	349(53.4)		
Education					
1	1,042(92.5)	421(94.8)	625(95.6)		
2	70(6.2)	23(5.2)	29(4.4)		
3	14(1.2)	0(0.0)	0(0.0)		
Self-report of health				65.470	0.000
Very good	64(5.68)	6(1.4)	10(1.5)		
Good	69(6.13)	10(2.3)	14(2.1)		
Fair	446(39.61)	143(32.2)	176(26.9)		
Poor	424(37.66)	193(43.5)	289(44.2)		
Very poor	123(10.92)	92(20.7)	165(25.2)		
Drink				5.746	0.003
No	577(51.24)	269(60.6)	360(55.0)		
Yes	549(48.76)	175(39.4)	294(45.0)		
Smoke				3.336	0.036
No	840(74.60)	354(79.7)	478(73.1)		
Yes	286(25.40)	90(20.3)	176(26.9)		
Contact with children in person/phone/email				3.818	0.022
No	140(12.43)	68(15.3)	64(9.8)		
Yes	986(87.57)	376(84.7)	590(90.2)		
Economic supports from children				0.458	0.633
No	141(12.52)	48(10.8)	77(11.8)		
Yes	985(87.48)	396(89.2)	577(88.2)		
Social activities				3.811	0.022
No	700(62.17)	308(69.4)	410(62.7)		
Yes	426(37.83)	136(30.6)	244(37.3)		
currently working				0.631	0.532
No	600(53.29)	244(55.0)	366(56.0)		
Yes	526(46.71)	200(45.0)	288(44.0)		
whether retired				0.416	0.660
No	545(48.40)	210(47.3)	302(46.2)		
Yes	581(51.60)	234(52.7)	352(53.8)		
Life satisfaction				58.825	0.000
Not at all satisfied	8(0.7)	42(9.5)	54(8.3)		
Not very satisfied	82(7.3)	72(16.2)	82(12.5)		
Somewhat satisfied	574(51.0)	194(43.7)	351(53.7)		
Very satisfied	405(36.0)	122(27.5)	147(22.5)		
Completely satisfied	57(5.1)	14(3.2)	20(3.1)		
Chronic diseases				1.141	0.320
No	81(7.2)	24(5.4)	38(5.8)		
Yes	1,045(92.8)	420(94.6)	616(94.2)		
Place of residence				7.348	0.001
Urban	322(28.60)	87(19.6)	157(24.0)		
Rural	804(71.40)	357(80.4)	497(76.0)		

### 3.5 Multinomial logistic of demographic variables for three latent profile regression results

A multinomial logistic regression analysis was conducted with gender, family location, frequency of meeting with children, financial support from children, social activities, chronic diseases, smoking, alcohol consumption, and life satisfaction as independent variables and potential categories of depression in older adults as dependent variables, and the results are shown in Table 5.

**Table 5:** Multinomial logistic regression of depression profiles.

Variables(ref)	Low level (ref) vs. high level depression		Low level (ref) vs. unstable affective depression		Unstable affective (ref) vs. high level depression	
	OR(95% CI)	*p*	OR(95% CI)	*p*	OR(95% CI)	*p*
Age, mean ± SD	1.01(0.989–1.031)	0.363	0.991(0.972–1.009)	0.315	1.019(0.997–1.043)	0.097
Gender (Female)						
Male	0.444(0.325–0.607)	0.000	0.684(0.524–0.893)	0.005	0.649(0.467–0.902)	0.010
Education (3)						
1	28323903.59(16756501.9–47876550.8)	0.000	46154446.9(28593430.35–74500783.66)	0.000	0.614(0.34–1.106)	0.104
2	37770559.52(37770559.52–37770559.52)		35243420.93(35243420.93–35243420.93)	.	1.072(1.072–1.072)	
Self-report of health (Very poor)						
Very good	0.127(0.05–0.321)	0.000	0.125(0.058–0.267)	0.000	1.014(0.343–2.995)	0.980
Good	0.226(0.107–0.479)	0.000	0.184(0.096–0.353)	0.000	1.23(0.509–2.971)	0.645
Fair	0.487(0.34–0.696)	0.000	0.335(0.245–0.458)	0.000	1.454(1.012–2.088)	0.043
Poor	0.651(0.464–0.914)	0.013	0.556(0.416–0.744)	0.000	1.171(0.845–1.623)	0.343
Drink (Yes)						
No	1.046(0.798–1.372)	0.743	1.042(0.822–1.32)	0.733	1.004(0.754–1.337)	0.977
Smoke (Yes)						
No	0.896(0.646–1.244)	0.513	0.79(0.603–1.035)	0.087	1.135(0.805–1.6)	0.469
Contact with children in person/phone/email (Yes)						
No	1.155(0.826–1.614)	0.400	0.755(0.542–1.051)	0.096	1.529(1.053–2.221)	0.026
Economic supports from children (Yes)						
No	0.808(0.554–1.176)	0.266	0.88(0.638–1.215)	0.438	0.917(0.617–1.363)	0.669
Social activities (Yes)						
No	1.289(1.005–1.654)	0.046	0.962(0.777–1.19)	0.719	1.341(1.029–1.746)	0.030
currently working (Yes)						
No	1.448(0.634–3.305)	0.380	1.137(0.545–2.372)	0.732	1.273(0.54–3.001)	0.581
whether retired (Yes)						
No	1.353(0.594–3.082)	0.471	1.094(0.526–2.276)	0.810	1.237(0.527–2.907)	0.625
Life satisfaction (Completely satisfied)						
Not at all satisfied	18.268(6.843–48.767)	0.000	14.185(5.639–35.682)	0.000	1.288(0.57–2.91)	0.543
Not very satisfied	3.447(1.735–6.849)	0.000	2.676(1.449–4.944)	0.002	1.288(0.6–2.765)	0.516
Somewhat satisfied	1.425(0.762–2.666)	0.268	1.774(1.029–3.059)	0.039	0.803(0.393–1.642)	0.548
Very satisfied	1.326(0.7–2.514)	0.386	1.143(0.651–2.005)	0.642	1.161(0.557–2.42)	0.691
Chronic diseases (Yes)						
No	1.043(0.621–1.752)	0.874	1.272(0.816–1.983)	0.289	0.82(0.469–1.432)	0.485
Place of residence (Rural)						
Urban	0.667(0.495–0.9)	0.008	0.846(0.659–1.087)	0.191	0.788(0.574–1.083)	0.142

OR, Odds ratio; 95%CI, 95% Confidence Interval.

As shown in Table 5, when comparing low and high levels of depression, gender (OR = 0.444, *p* = 0.000), primary education (OR = 28323903.59, *p* = 0.000), self-assessed health (OR = 0.127, *p* = 0.000; OR = 0.226, *p* = 0.000; OR = 0.487, *p* = 0.000; OR = 0.651, *p* = 0.013), participation in social activities (OR = 1.289, *p* = 0.046), complete or poor life satisfaction (OR = 18.268, *p* = 0.000; OR = 3.447, *p* = 0.000), and place of residence (OR = 0.667, *p* = 0.008) were the influential high level depressive type factors; when comparing low level depression and unstable affective depression, gender (OR = 0.684, *p* = 0.005), primary education (OR = 46154446.9, *p* = 0.000), self-assessed health (OR = 0.125, *p* = 0.000; OR = 0.184, *p* = 0.000; OR = 0.335, *p* = 0.000; OR = 0.556, *p* = 0.000), complete or poor or fair life satisfaction (OR = 14.185, *p* = 0.000; OR = 2.676, *p* = 0.002; OR = 1.774, *p* = 0.039) were the influencing factors of unstable affective depressive type. Gender (OR = 0.649, *p* = 0.010), average self-assessed health (OR = 1.454, *p* = 0.043), weekly communication with children (OR = 1.529, *p* = 0.026), and participation in social activities (OR = 1.341, *p* = 0.030) were influential factors for high level depressive type when unstable affective depressive type was used as a reference.

## 4 Discussion

The purpose of this study was to delineate depressive subgroups of older adults spouse caregivers and to explore the factors influencing depression. We rationalized the selection of three profiles and named them as low-level depressive, high-level depressive, and unstable affective depressive.

The results of the study showed that about 50.6% of the older adult’s spouse caregivers were classified as low-level depressive type. Their lower mean scores on the scale items indicate relatively low levels of overall depression, with higher scores observed only on specific items. For example, “My sleep was restless.” and “I felt hopeful about the future.” the former may be because older adults’ sleep decreases with age (41), while the latter may be because older spouse caregivers often suffer from emotional fatigue, they need to focus on caring for their spouse at the expense of their own health (42), and they shoulder more responsibilities and thus feel hopeless about the future. However, despite the fact that this subgroup of older adults exhibits a low level of depressive type, we should not ignore their needs. They still need to receive interventions in specific areas in order to avoid the transition to high levels of depression. In univariate analyses, we found that older adults in this subgroup performed best in terms of self-assessed health status, life satisfaction, and frequency of social participation, which explains the reason for our groups of them.

20.4% of older spouse caregivers were classified as high level depressed, with essentially the highest mean scores on each of the three profiles. In this subgroup, the item “I feel scared” had the highest score, which means that this group of older spouse caregivers not only had higher levels of depression overall, but also had the most prominent feeling of fear. In addition, this group had lower life satisfaction, higher rates of illness, and the lowest frequency of social participation and communication with their children. As caregivers, they worry not only about their own health, but also about their inability to care for their spouses. For this group of older adults, health education and targeted interventions to alleviate their depression are critical. These interventions can focus on their physical health as well as provide support and guidance to help them effectively care for their spouse.

29.4% of the older spouses were classified as unstable depressed. The most significant characteristic of this group of older spouse caregivers is the instability of depressed mood, and they fluctuate more in the expression dimension of depressed mood. The item “I felt depressed.” had the highest score, while the item “I felt fearful” had the lowest score. According to Figure 1, the depression level of this category of older adult’s caregivers is similar to that of the high level depressed type, but their mood swings significantly exceed those of the high level depressed type. Although this group of older adults had lower levels of education and poorer self-rated health, they had the highest percentage of communication with their children. Therefore, this result is likely to be related to the effective support they receive from their children. It has also been confirmed that frequent communication with children can be effective in alleviating depression levels in older adults (43, 44).

Our study found that gender, education level, self-health assessment, communication with children, social participation, life satisfaction, and residence were influential factors of depression in older adults spouse caregivers. Specifically, education level, life satisfaction were significant when low level depression type was compared with high level depression type and unstable affective depression type. All samples or different age groups, education was significantly positively associated with depression (45). Satisfaction with life reduced the occurrence of depression to some extent (46). Life satisfaction reflects older adults’ overall contentment with various aspects of their lives, and when they experience dissatisfaction or face difficulties, they may be more prone to depressive moods. Place of residence situation is meaningful when low level depression type is compared with high level depression type. Urban older adults have a lower prevalence of depression than rural older adult (46). This may be related to the mental health protective effects of urbanization, where urban environments provide better infrastructure, resources, opportunities, and improved social support and benefits (47, 48). Both are meaningful when unstable affective depressive social activity participation and weekly interaction with children are compared with high levels of depressive social activity participation and weekly frequency of interaction with children. Social activity is a significant influence on depressive symptoms in older adults (43), and older adults who are less socially active are more likely to experience depressive symptoms (46). Older adults who maintain weekly contact with their children are less likely to have depression (44). Both participation in activities, interaction with others during activities, and emotional comfort from children can provide older adults with a sense of presence and satisfaction, thus reducing the level of depression in older age groups. In Chinese culture, people often express their love and longing for their children implicitly, especially among the older adults, who often worry about adding burdens to their children (49). Therefore, the state can help alleviate depression in older spousal caregivers by encouraging communication between children and parents, promoting social engagement among older adults, and preventing the transition from unstable affective depression to severe depression.

Studies have demonstrated that self-rated health is a significant factor that influences depression in older adults (50). These caregivers may be more concerned about their health status because of differences in their lifestyles from other older adults. They may be more concerned about their own health and fear that no one will care for them, and more so, that they will not be able to care for their spouse. Furthermore, individuals with more favorable self-reports of their health status tend to have a confident outlook on life and are less likely to experience depression (31). More interestingly, depression is also a predictor of self-assessment of health (51). This is understandable, as higher self-assessed health is associated with more positive mood. Moreover, when individuals have higher self-reports of their health status, it indicates that they are confident in their health and more likely to adopt a positive attitude toward life (43), and thus less likely to be depressed.

Gender is also an influencing factor for depression in older adults (46). Compared to men, women are more likely to fall into depressive moods. This phenomenon may stem from the fact that women themselves often have to take on more domestic tasks, and when they also have the responsibility of caring for their spouses, the load increases, which triggers more severe depressive situations. In addition, many of the other women live more dependent on men, have less social contact, and are less involved in activities, which may also contribute to differences in depressive conditions between men and women (46, 52). Participation in social activities has a positive effect on the mental health of older adults. However, reduced social activities due to long-term caregiving roles may also contribute to increased levels of depression. Therefore, we should pay more attention to the depressive status of older women, provide them with more help, care and social support, and encourage them to participate more in social activities and maintain communication with their children.

### 4.1 Limitations

This study has several limitations that should be considered. Firstly, the CES-D-10 instrument used is a screening tool that is not definitive in identifying depression in older spouse caregivers. Secondly, the data was gathered through self-reporting, which may be vulnerable to some degree of bias. Lastly, the study utilized a cross-sectional design that did not permit the establishment of causal relationships between the findings.

## 5 Conclusion

Our study grouped depression in older spouse caregivers into three subgroups, each presenting different characteristics. Such a division provided us with a deeper understanding of depression among older adults spouse caregivers. To our knowledge, this is the first study to apply LPA to analyze depression in older adults spouse caregivers in China. Our findings suggest that depression among older spouse caregivers is influenced by several factors, including: gender, education, self-assessment of health, communication with children, social participation, life satisfaction, and place of residence. By comparing subgroups, we can identify populations disproportionately affected by depression and develop targeted interventions accordingly. Moving forward, implementing tailored support programs based on the distinct depression profiles of older spousal caregivers through primary healthcare systems will be critical. Given that older adults already face elevated depression risks, spousal caregivers constitute a particularly vulnerable subgroup due to their dual roles of advanced age and intensive caregiving responsibilities. This confluence of factors creates an urgent need for specialized psychological interventions. Healthcare strategies should simultaneously address both the manifest psychological distress and its root causes, while policymakers must establish integrated support systems to foster healthy aging trajectories for this population.

## Data Availability

The raw data supporting the conclusions of this article will be made available by the authors, without undue reservation.
